# Spatial distribution of traditional male circumcision and associated factors in Ethiopia; using multilevel generalized linear mixed effects model

**DOI:** 10.1186/s12889-021-11482-5

**Published:** 2021-07-19

**Authors:** Biruk Shalmeno Tusa, Adisu Birhanu Weldesenbet, Telahun Kasa Tefera, Sewnet Adem Kebede

**Affiliations:** 1 Department of Epidemiology and Biostatistics, College of Health and Medical Sciences, Haramaya University, Haramaya, Ethiopia; 2 Department of Nursing, Dessie Health Science College, Dessie, Ethiopia; 3 Department of Epidemiology and Biostatistics, College of Medicine and Health Sciences, Institute of Public Health, University of Gondar, Gondar, Ethiopia

**Keywords:** Traditional male circumcision, Spatial analysis, Multilevel generalized linear mixed effects model, Ethiopia

## Abstract

**Background:**

Traditional male circumcision (TMC) is primarily associated with a religious or cultural purpose and may lead to complications. To reduce risks of complication and long-term disabilities that may happen from circumcisions that are undertaken in non-clinical settings, information concerning TMC is very important. Therefore, this study is aimed at identifying spatial distribution of TMC and the factors associated with TMC in Ethiopia.

**Methods:**

A secondary data analysis was conducted among 11,209 circumcised males using data from 2016 Ethiopian Demographic and Health Survey (EDHS). Global Moran’s I statistic was observed to check whether there was a significant clustering of TMC. Primary and secondary clusters of TMC were identified by fitting Bernoulli model in Kulldorff’s SaTScan software. Multilevel Generalized Linear Mixed effects Model (GLMM) was fitted to identify factors associated with TMC.

**Result:**

The spatial distribution of TMC was nonrandom across the country with Global Moran’s I = 0.27 (*p*-value < 0.0001). The primary clusters of TMC were identified in the southern part of Oromia and Tigray, northern part of SNNPR, Amhara, Gambella and Benishangul regions. Current age, age at circumcision, ethnicity, religion, place of residence, wealth index, media exposure, sex of household head and age of household head were factors associated with TMC in Ethiopia.

**Conclusions:**

The spatial distribution of TMC was varied across the country. This variation might be due to the diversity of culture, ethnicity and religion across the regions. Thus, there is a need to rearrange the regulations on standards of TMC practice, conduct training to familiarize operation technique and general hygiene procedures, and launch cross-referral systems between traditional circumcisers and health workers. While undertaking these public health interventions, due attention should be given to the identified clusters and significant factors.

## Background

Traditional male circumcision (TMC) refers to the procedure of performing male circumcision on adolescents or young male in a non-clinical setting by a traditional provider with no formal medical training. It is usually associated primarily with a religious or cultural ceremony. There is great variation across nations at what age circumcision has to be performed. It is performed on the eighth birth day among Jewish male infants, yet, among others, it occurs at any age between birth and puberty [1, 2].

Studies from Africa suggest that male circumcision (MC) reduces human immunodeficiency virus (HIV) acquisition in heterosexual male by 50–60% [3–5]. It is recommended as HIV prevention program by the World Health Organization (WHO) and United Nations (UN) considering its effectiveness in reducing HIV by around 60% among males. Accordingly, MC should be performed on infant and adult males up to 35 years of age. Early infant MC is more effective in terms of cost and makes adverse events less likely [6–8].

The projected percentage of circumcised males in each country and territory varies significantly. Based on 2015 US Central Intelligence Agency (CIA) data, global MC prevalence was 38.7% [9]. Approximately half of circumcisions were for religious and cultural reasons [9]. In Africa, the magnitude of TMC shows great variation across countries and even within the same country-i.e. between urban and rural areas, with reported prevalence of 20, 37 and 80% from Uganda, southern Africa, and Kenya respectively [2, 10].

Traditional male circumcision is associated with both short-term and long-term complications. The overall TMC related complication rates of 35 and 48% were reported by circumcised male in Kenya and South Africa respectively [11, 12]. Wound infection, severe hemorrhage, retention of urine, swelling and amputations of the glans penis are most frequent complications encountered by male undergoing traditional circumcision [13, 14].

Thromboembolism, dehydration and congestive heart failure are common complication of traditionally circumcised men [15]. Around 86% of those undergoing traditional circumcision also reported severe pain. The long-term adverse sequence include loss of erectile function, persistent swelling, extensive scarring, complete or partial amputation of the penis which accounts for around 14% hospital admissions after traditional male circumcision [11, 12].

To reduce risks of complication and long term disabilities that may happen from circumcisions that are undertaken in non-clinical settings information concerning TMC is of paramount importance in Sub-Saharan Africa where the problem of TMC is worse [10]. Knowing spatial distribution of TMC is equally important in overcoming its adverse events to prioritize intervention in areas with high clusters of TMC.

Despite the above fact, little is known about factors affecting TMC in resource limited settings such as Ethiopia. Moreover, to the best of authors’ knowledge, this is the first study to assess geographical variation and factors affecting TMC in Ethiopia. Therefore, this study is aimed at identifying spatial distribution of TMC and its associated factors in Ethiopia by applying spatial analysis and linear mixed effect model.

## Methods

### Study setting

This study was conducted in Ethiopia, which is located in the horn of Africa. The country has nine regions (Afar, Tigray, Amhara, Oromia, Somali, Southern Nations, Nationalities, and People’s Region (SNNPR), Benishangul Gumuz, Gambella and Harari) and two administrative cities (Addis Ababa and Dire Dawa). Except Harari and the two administrative cities (Addis Ababa and Dire Dawa), the rest of regions are predominantly rural. The high proportion of Christians are found in Addis Ababa, Amhara, Tigray, Southern Nations and Nationalities Peoples Region, and Gambella region, while Islam is most prevalent in the Afar, Harari and Somali Regions.

### Data source

The source of data for this study was the 2016 Ethiopia demography and heath survey (EDHS). Data was accessed after permission was approved by major demographic and health survey through an online request at http://www.dhsprogram.com.The 2016 EDHS was the fourth Demographic and Health Survey, which was conducted using a two-stage stratified cluster sampling method from January 18, 2016, to June 27, 2016 [16]. In the first stage, a total of 645 Enumeration Areas (EAs) (443 in rural areas and 202 in urban areas) were taken with probability proportional to EA size and with independent selection in each sampling stratum. In the second stage of selection, a fixed number of 28 households per cluster were selected with an equal probability systematic selection from the newly created household listing [16]. In the present study, the total weighted samples of 11,209 circumcised male were taken. Latitude and longitude coordinates had been also included from selected EAs (clusters). The detailed sampling system were found in the full EDHS report [16].

### Dependent and independent variables

The dependent variable of the present study was traditional male circumcision (TMC). The 2016 EDHS asked the circumcised male to answer the question “who performed the circumcision”. If the circumcision was performed by a traditional circumciser or family or friends or other traditional, it was coded as “Yes = 1”. In contrast, if the circumcision was performed by a doctor, a nurse, or other health professional, it was coded as “No = 0”. The response variable of the i^th^ individual Yi was categorized as a dichotomous variable with possible values Yi = Yes, if i^th^ the individual had experienced traditional circumcision and Yi = No, if the individual did not experience traditional circumcision. The independent variables included in the analysis are current age, age at circumcision, religion, ethnicity, place of residence, wealth index, media exposure (frequency of reading magazine or newspaper, frequency of listening radio and frequency of watching Television), age of household head and sex of household head. In DHS, the wealth index is calculated using easy-to-collect data on a household’s ownership of selected assets, such as televisions and bicycles; materials used for housing construction; and types of water access and sanitation facilities.

### Data processing

STATA 14; ArcGIS 10.1 and SaTScan 9.6 software’s were used to perform data analysis. Any statistical analysis was performed after the data was weighted using sampling weight (men’s sample weight), primary sampling unit, and strata. This enables to restore the representativeness of the survey and to communicate the STATA to consider the sampling design when computing standard errors which helps to find unbiased statistical estimates. Descriptive and summary statistics were reported in form of text, figures and tables.

### Statistical analysis

#### Spatial analysis

To determine whether there was a significant clustering of TMC, the spatial autocorrelation (Global Moran’s I) statistic was observed. Moran’s I have a value ranging from-1 to 1. Clustered TMC is confirmed when Moran’s I value is positive while negative Moran’s I shows that TMC is dispersed. When the value of Moran’s I is near zero, it is concluded that traditional male circumcision is randomly distributed. In addition, both Z-score and *P*-value are generated to measure the significance of the Moran index. Getis-Ord Gi* statistics was used for hot-spot analysis.

Using Kulldorff’s SaTScan software, Bernoulli based model was fitted to identify significant primary (most likely) and secondary clusters of TMC. The SaTScan applies a circular scanning window that moves across the study area. To fit the Bernoulli model, male who were circumcised traditionally were considered as cases, whereas those who were not circumcised traditionally were taken as controls. The default maximum spatial cluster size of < 50% of the population was considered as an upper limit, which permitted both small and large clusters to be identified yet clusters that contained more than the maximum limit be ignored. Areas with high log likelihood ratio and significant *p*-value were taken as areas with high TMC compared to areas outside of the window. The Kriging spatial interpolation method was also applied to forecast the un-sampled/unmeasured values from the sampled measurements.

#### Multilevel generalized linear mixed model

The probability of experiencing TMC varies across clusters; male within a cluster may be more similar to each other than male in other clusters. Due to this fact, the assumption of independence of observations and equal variance across clusters may be violated. To accommodate this, a cluster level random intercept is introduced in the generalized linear mixed model. Assume that *y*_*ij*_: the binary outcome for *i*^*th*^ individual in *j*^*th*^ cluster *j*, and assume *y*_*ij*_ follows Bernoulli distribution with probability of experiencing TMC (*p*_*ij*_)_._ Then, using the usual logit link function, a dichotomous outcome can be linked with linear predictor as follows: -.

*logit* (*p*_*ij*_) = *β*_0_ + *βx*_*ij*_ + *u*_*j*_ ; where *β*_0_ is an intercept, *βx*_*ij*_ is unkown parameter for individual level predictors, and *u*_*j*_ are mutually independent gaussian random effects used to capture within cluster correlation. To test the significance of the variance of random intercept, the likelihood ratio test was applied. Measures of variability for random effect were determined by computing Intra-class correlation coefficient (ICC). In the multivariable model adjusted odds ratio (AOR) with a 95% confidence interval (CI) and *p*-value 0.05 was considered to declare associated factors of traditional male circumcision.

## Results

### Characteristics of study population

**Fig. 1:**
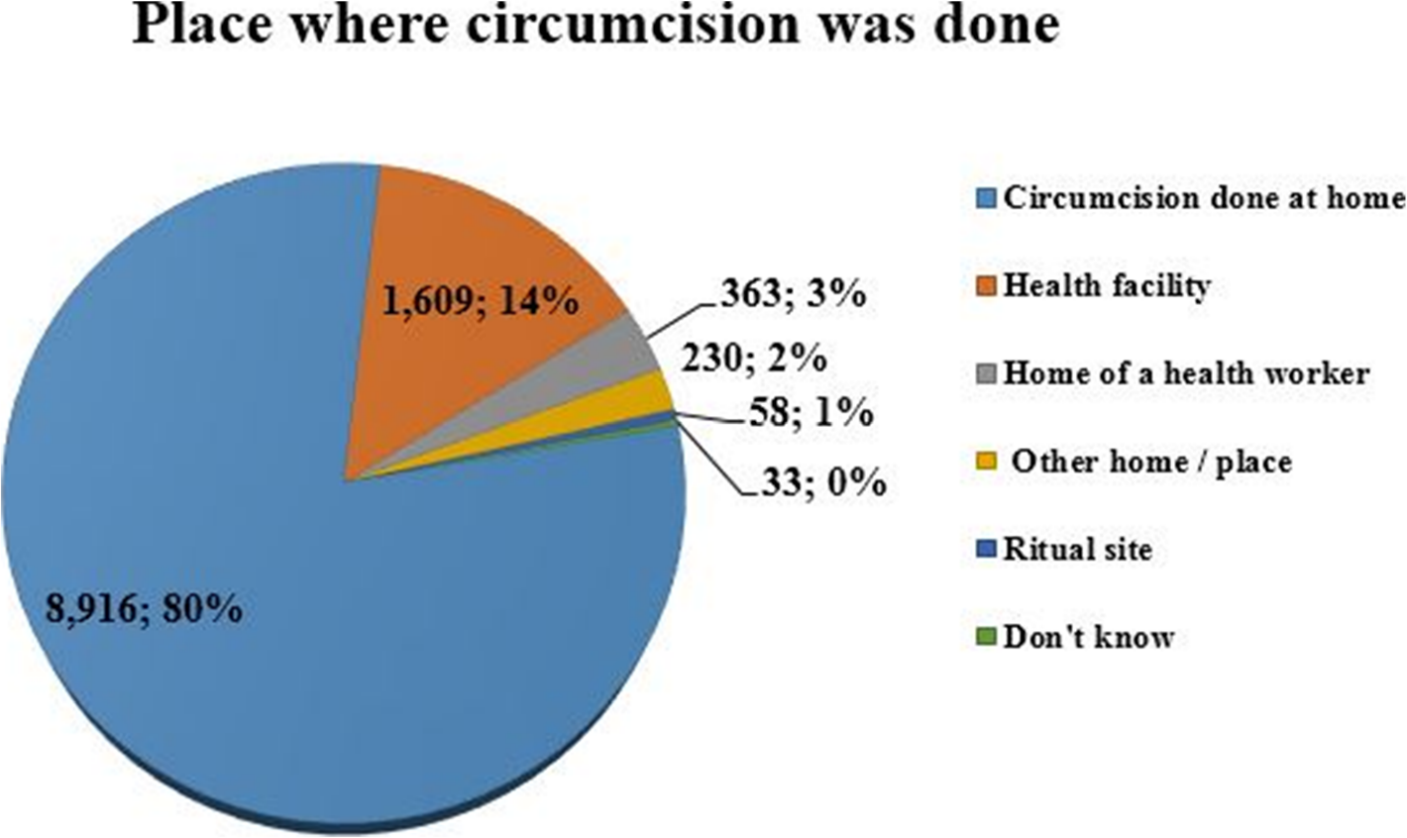
shows place where circumcision was done for study participants

A total of weighted samples of 11,209 circumcised male were taken in the analysis. Among these, 9205 (82.12%) were circumcised traditionally. Regarding the place where circumcisions were performed, 80 % of them were done at home whereas the rest 14 % took place at health facility (Fig. 1**)**. A near to two-thirds (66.06%) of respondents were circumcised before age of 5 years.

Among 11,209 circumcised male, 2122 (18.93%) of them were under the age of 20 years, 5099 (45.49%) of them were Orthodox Christians and 2240 (19.99%) of them were rural residents. More than one-third (36.51%) of the study participants were ethnic Oromo and more than one quarter (25.38%) of them were richest in wealth index. Concerning media exposure, more than one-third of the respondents did not have media exposure (Table 1).

**Table 1:** Shows Characteristics of study population in Ethiopia from January 18 to June 27, 2016 (*N* = 11,209)

Variables	Traditional circumcision (Weighted)		Total (Weighted)	Percent
	Yes	No		
**Current age**				
15–19	1572	550	2122	18.93
20–24	1250	386	1636	14.60
25–29	1379	362	1741	15.53
30–34	1190	279	1469	13.11
35–39	1112	167	1279	11.41
40–44	970	110	1090	9.63
45–49	808	70	878	7.84
50–54	499	48	547	4.88
55–59	425	32	457	4.07
**Age at circumcision**				
0–4	6922	483	7405	66.06
5–9	922	230	1152	10.27
10–14	694	488	1182	10.55
15–19	295	558	853	7.61
20–24	42	120	162	1.45
Above 25	33	37	70	0.63
Don’t know	297	88	385	3.43
**Religion**				
Orthodox	4545	554	5099	45.49
Muslim	3175	542	3717	33.17
Protestant	1347	862	2209	19.71
Catholic	60	15	75	0.66
Traditional	17	2	19	0.17
Other	61	29	90	0.80
**Ethnicity**				
Oromo	3441	651	4092	36.51
Amhara	3202	261	3463	30.90
Tigrie	725	39	764	6.81
Sidama	142	287	429	3.83
Somalie	304	20	324	2.89
Guragie	261	52	313	2.79
Afar	61	6	67	0.60
Other	1069	688	1757	15.68
**Place of residence**				
Urban	1769	471	2240	19.99
Rural	7436	1533	8969	80.01
**Wealth index**				
Poorest	1500	212	1712	15.27
Poorer	1738	274	2012	17.95
Middle	1796	376	2172	19.37
Richer	1982	486	2468	22.03
Richest	2189	656	2845	25.38
**Media exposure**				
No	3364	591	3955	35.29
Yes	5841	1413	7254	64.71
**Sex of household head**				
Male	8215	1648	9863	87.98
Female	990	356	1346	12.02
**Age of household head**				
15–19	70	8	78	0.70
20–24	391	75	466	4.16
25–29	1026	254	1280	11.42
30–34	1198	235	1433	12.79
35–39	1255	245	1500	13.39
40–44	1280	188	1468	13.10
45–49	1186	198	1384	12.35
Above 49	2799	801	3600	32.11
**Total**	**9205**	**2004**	**11,209**	**100%**

### Spatial analysis of traditional male circumcision

**Fig. 2:**
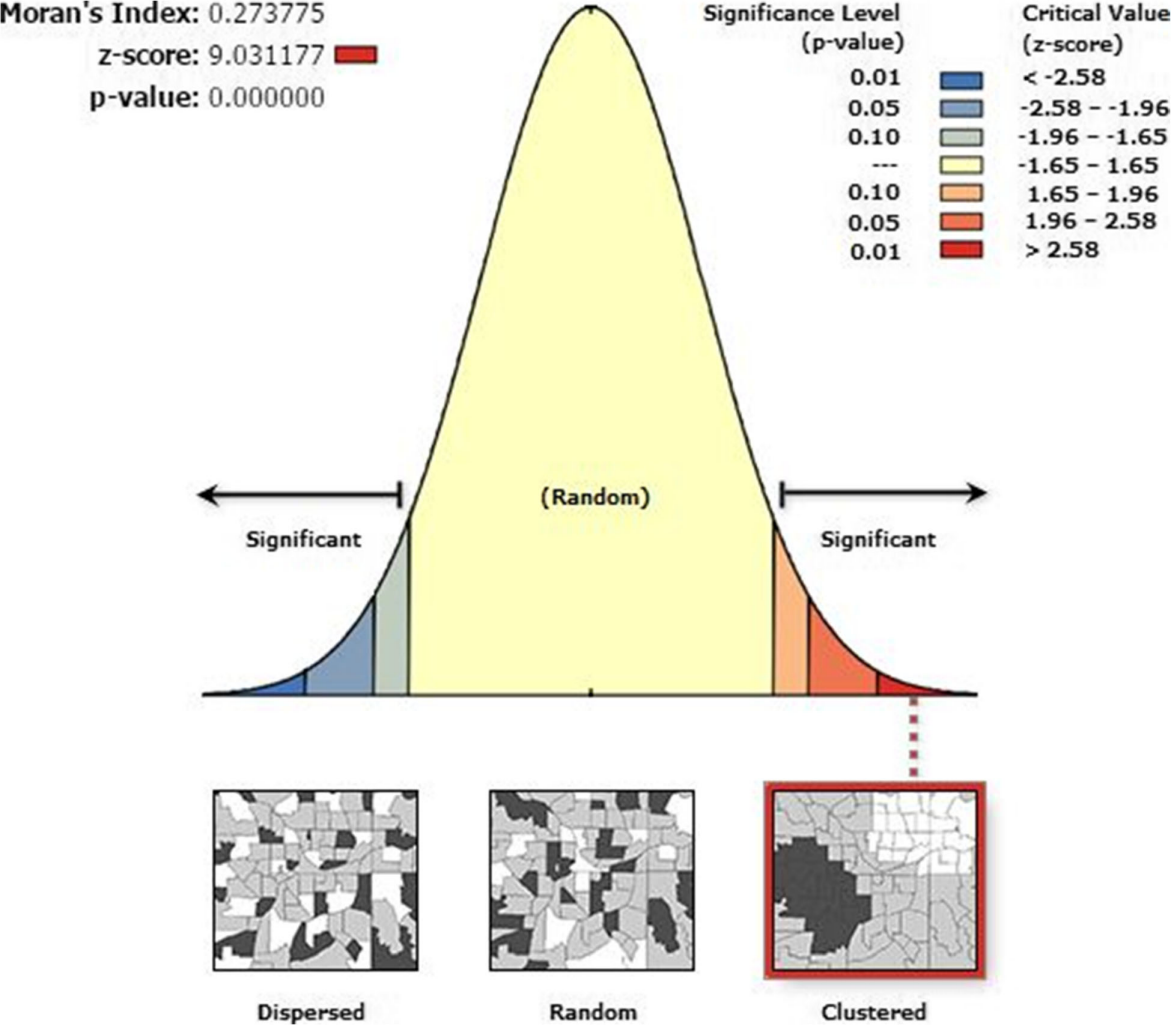
Spatial autocorrelation analysis of traditional male circumcision in Ethiopia, 2016

**Fig. 3:**
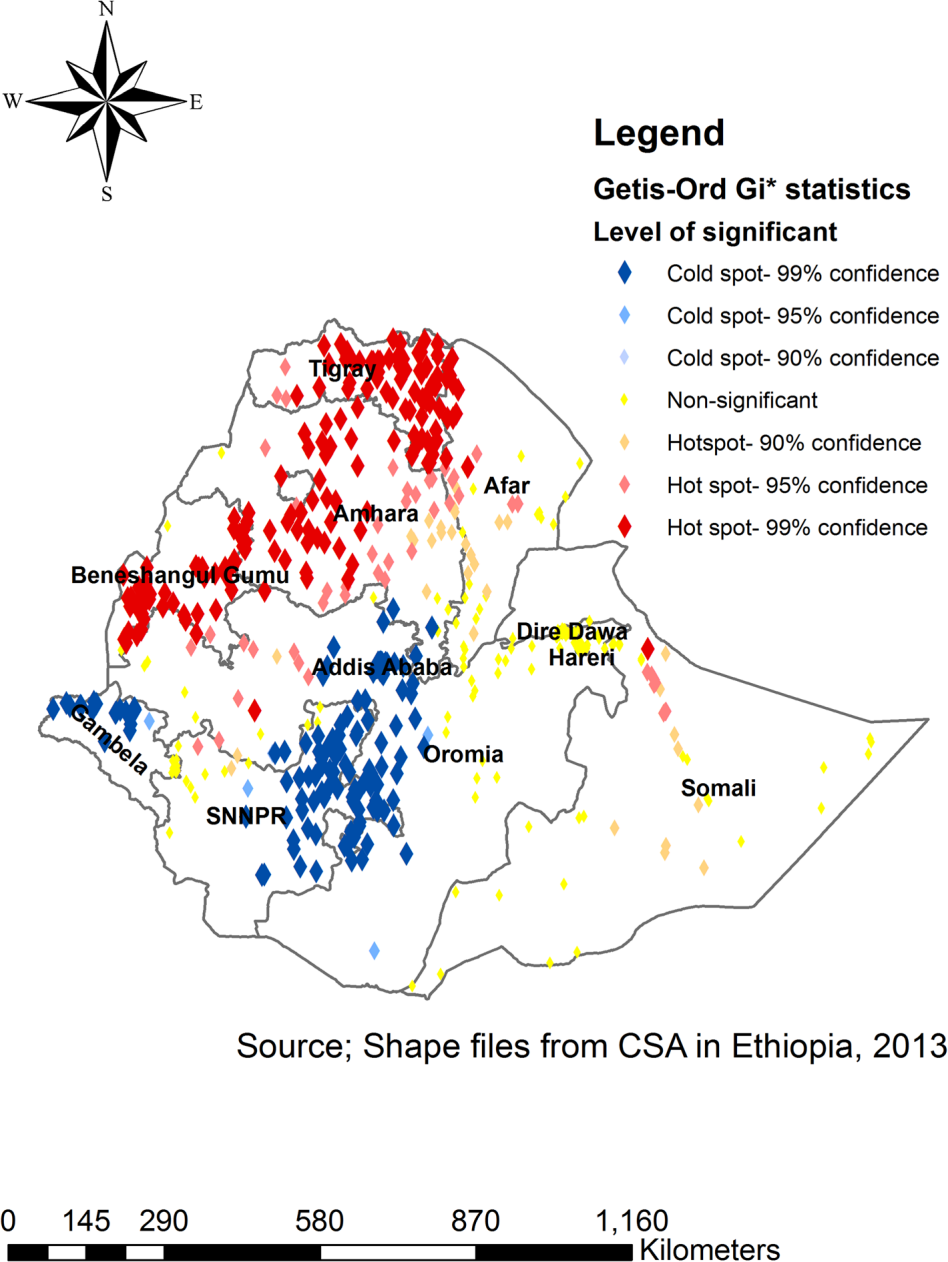
Hotspot and Cold areas of traditional male circumcision across regions in Ethiopia, 2016

The spatial distribution of traditional male circumcision in Ethiopia was varied across the country with Global Moran’s I = 0.27 (*p*-value < 0.0001) (Fig. 2). The Getis Ord Gi statistical analysis displays the hot-spot and cold-spot areas of TMC in Ethiopia. The red colors indicate the significant hotspot areas (higher proportion TMC), which were identified in Tigray, Amhara, west part of Afar and Benishangul regions. In contrast, the blue color shows significant cold-spot areas (areas with low TMC), which were observed in Gambella, Addis Ababa, Eastern part of SNNPR and central part of Oromia regions (Fig. 3).

**Fig. 4:**
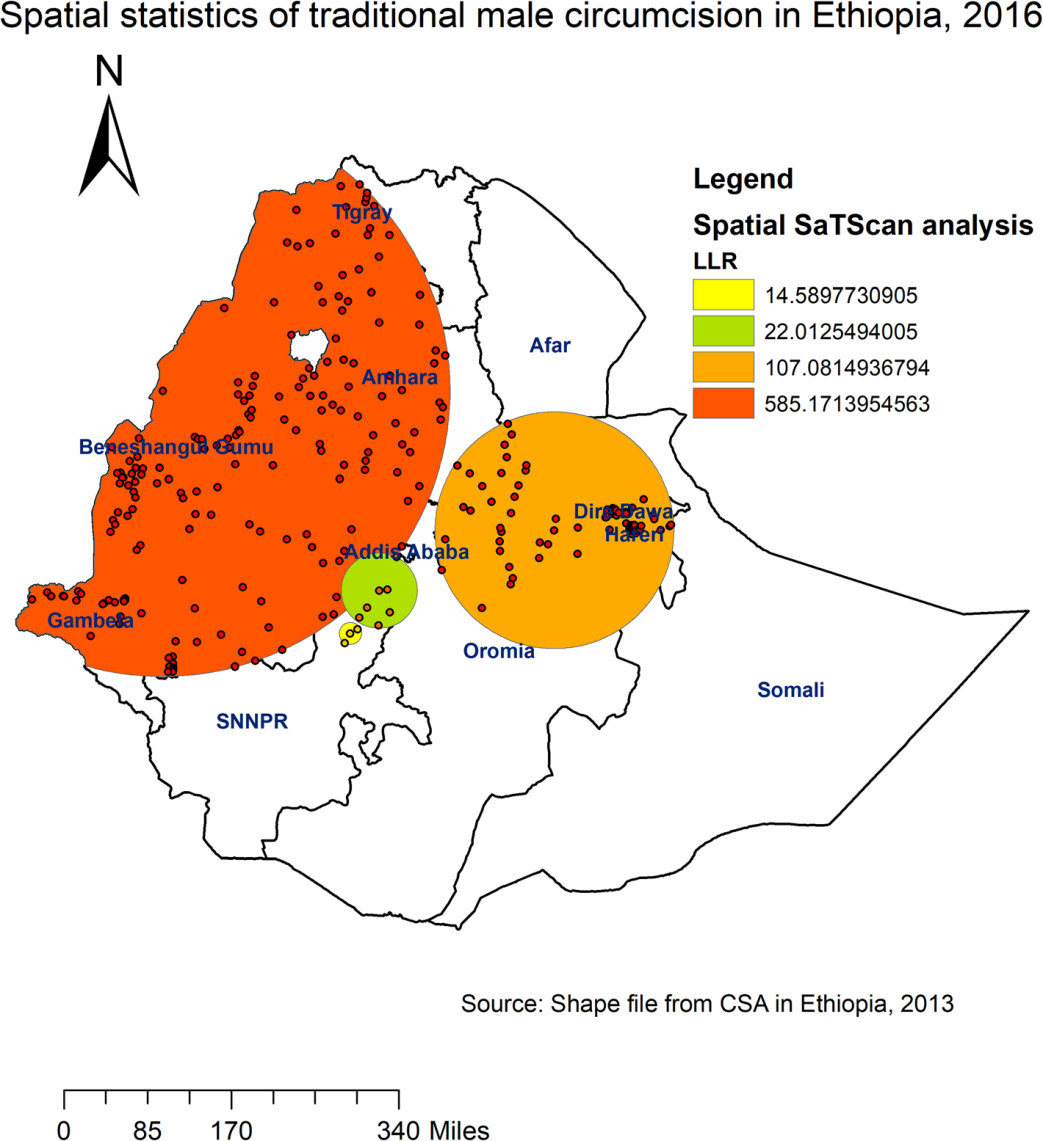
Primary and secondary clusters of traditional male circumcision across regions in Ethiopia, 2016

**Fig. 5:**
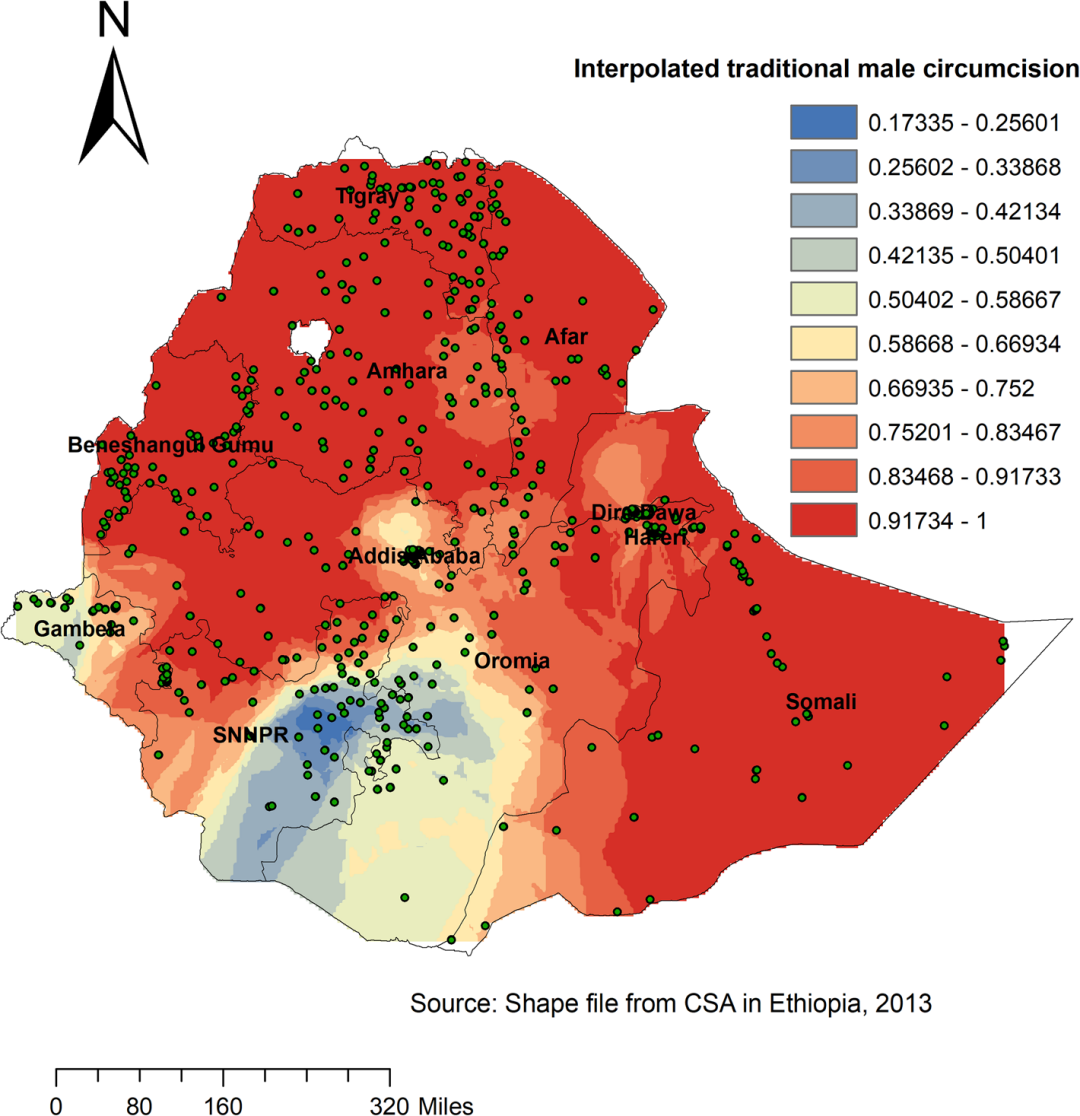
Kriging interpolation of traditional male circumcision across regions in Ethiopia, 2016

According to the Spatial SaTScan analysis (using Bernoulli based model), total of 384 significant primary (shaded by red colour in Fig. 4) and secondary clusters (shaded by orange colour in Fig. 4) of TMC were observed. From these, 188 were primary clusters. The primary clusters were identified in the southern part of Oromia and Tigray, Amhara, northern part of SNNPR, Gambella and Benishangul regions at 11.34 N, 35.13 E with 467.9 km radius, a Relative Risk (RR) of 1.33, and Log-Likelihood Ratio (LRR) of 585, at *p*-value < 0.001. This revealed that male within the spatial window had 1.33 times higher chance of undergoing TMC as compared to those male outside the spatial window (Fig. 4). In Kriging interpolation, the highest prevalence of TMC was observed in Tigray, Amhara, Benishangul, and Somalia regions, whereas relatively low prevalence of TMC was detected in eastern part of SNNPR, central Gambella, western part of Oromia and Addis Ababa (Fig. 5).

### Factors associated with traditional male circumcision

In Multilevel Generalized Linear Mixed Model, age, age at circumcision, place of residence, media exposure, sex of household head, age of household head were significant variables(Table 2). The odds of traditional male circumcision were higher among male aged 20–59 than male aged 15–19 years. However, the likelihoods of traditional male circumcision were low among male who were circumcised after the age of five as compared to male who were circumcised before the age of five. The odds of traditional male circumcision decreased by 39 and 61% among orthodox and protestant male respectively as it is compared with Muslim male.

**Table 2:** Associated factors of traditional male circumcision determined by Multilevel Generalized Linear Mixed Model

Variables	Odds Ratio (95% CI)		***P***-value
	COR	AOR	
**Current age**			
15–19	1	1	–
20–24	1.51 [1.23, 1.86]	1.58 [1.26, 1.98]	< 0.001***
25–29	1.97 [1.60, 2.44]	1.77 [1.36, 2.29]	< 0.001***
30–34	2.62 [2.07, 3.32]	2.26 [1.67, 3.05]	< 0.001***
35–39	3.93 [3.02, 5.11]	3.65 [2.64, 5.05]	< 0.001***
40–44	7.74 [5.66, 10.59]	7.26 [5.04, 10.44]	< 0.001***
45–49	11.90 [8.05, 17.61]	13.91 [8.76, 22.10]	< 0.001***
50–54	9.08 [5.94, 13.89]	14.34 [8.90, 23.30]	< 0.001***
55–59	13.74 [8.13, 23.21]	20.34 [11.29, 36.63]	< 0.001***
**Age at circumcision**			
0–4	1	1	–
5–9	0.45 [0.37, 0.55]	0.43 [0.35, 0.54]	< 0.001***
10–14	0.23 [0.19, 0.29]	0.20 [0.16, 0.25]	< 0.001***
15–19	0.09 [0.07, 0.12]	0.06 [0.04, 0.08]	< 0.001***
20–24	0.06 [036, 0.11]	0.03 [0.02, 0.06]	< 0.001***
Above 25	0.15 [0.07, 0.30]	0.06 [0.03, 0.13]	< 0.001***
Don’t know	0.96 [0.71, 1.30]	0.82 [0.60, 1.13]	0.235
**Religion**			
Muslim	1	1	–
Orthodox	0.58 [0.47, 0.71]	0.61 [0.48, 0.78]	< 0.001***
Protestant	0.26 [0.20, 0.34]	0.39 [0.29, 0.53]	< 0.001***
Catholic	0.61 [0.26, 1.42]	1.03 [0.37, 2.86]	0.962
Traditional	0.51 [0.09, 3.02]	0.38 [0.05, 3.02]	0.363
Other	0.33 [0.15, 0.74]	0.44 [0.18, 1.05]	0.065
**Ethnicity**			
Oromo	1	1	
Amhara	0.94 [0.74, 1.19]	0.89 [0.69, 1.15]	0.373
Tigrie	1.94 [1.30, 2.89]	1.99 [1.32, 3.01]	0.001**
Sidama	0.12 [0.05, 0.28]	0.11 [0.05, 0.24]	< 0.001***
Somalie	2.37 [1.50, 3.75]	1.66 [1.03, 2.65]	0.036*
Guragie	1.18 [0.84, 1.66]	1.01 [0.71, 1.45]	0.941
Afar	4.85 [2.46, 9.55]	2.48 [1.25, 4.92]	0.010*
Other	0.56 [0.42, 0.74]	0.74 [0.54, 1.01]	0.057
**Place of residence**			
Urban	1	1	
Rural	6.22 [4.24, 9.12]	3.80 [2.50, 5.76]	< 0.001***
**Wealth index**			
Poorest	1	1	
Poorer	0.67 [0.49, 0.92]	0.75 [0.52, 1.08]	0.120
Middle	0.57 [0.41, 0.78]	0.74 [0.52, 1.05]	0.093
Richer	0.47 [0.34, 0.64]	0.57 [0.40, 0.82]	0.002**
Richest	0.27 [0.19, 0.36]	0.43 [0.29, 0.64]	< 0.001***
**Media exposure**			
No			
Yes	0.50 [0.41, 0.61]	0.67 [0.54, 0.83]	< 0.001***
**Sex of household head**			
Male			
Female	0.48 [0.40, 0.56]	0.72 [0.60, 0.87]	0.001**
**Age of household head**			
Above 49			
15–19	1.42 [0.75, 2.70]	2.76 [1.41, 5.41]	0.003**
20–24	1.51 [1.10, 2.06]	2.54 [1.78, 3.63]	< 0.001***
25–29	1.53 [1.22, 1.91]	1.86 [1.41, 2.46]	< 0.001***
30–34	1.63 [1.31, 2.04]	1.78 [1.32, 2.39]	< 0.001***
35–39	1.63 [1.31, 2.04]	1.39 [1.05, 1.85]	0.023*
40–44	2.19 [1.74, 2.76]	1.64 [1.22, 2.20]	0.001**
45–49	1.97 [1.56, 2.49]	1.45 [1.09, 1.95]	0.012*

*AOR* Adjusted odd ratio, *CI* Confidence interval, *COR* Crude odd ratio; *: *P* < 0.05; **P < 0.005; ***P < 0.001

Ethnicity of study participants was found to be significantly associated with traditional male circumcision in Ethiopia. Afar, Tigrie and Somali male were 2.48, 1.99 and 1.66 times more likely to be circumcised by traditional methods respectively than Oromo male whereas the likelihood of traditional male circumcision decreased by 91% among Sidama male than Oromo male. Rural male were 3.80 times more likely to be circumcised by traditional methods than urban male were. The odds of traditional male circumcision decreased by 43 and 57% among richer and richest male as it compared with poorest male. The likelihoods of traditional male circumcision decreased by 33% among media exposed male as compared to their counterparts.

The sex and age of household head were also other factors that were significantly associated with traditional male circumcision in Ethiopia. The odds of traditional male circumcisions decreased by 52% among male who came from a household whose head is female than a household headed by male. Male who lived in a household whose head is lower than 49 years of age were more likely to be circumcised by traditional methods than their counterparts.

## Discussion

The present study reported that the spatial distribution of traditional male circumcision was clustered and primary cluster of TMC was observed in the southern part of Oromia, Tigray, Amhara, northern part of SNNPR, Gambella and Benishangul regions. The Kriging interpolation has also detected the highest prevalence of TMC in Tigray, Amhara, Benishangul, and Somalia regions. This variation might be due to the diversity of culture, ethnicity and religion across the regions. In Ethiopia, there are different religions and more than 80 languages. Most of the time, male circumcision is performed for cultural purpose, as an initiation ritual and a rite of passage into manhood [10]. This cultural identity and the crave to proceed ethnic conventions are the most grounded supporters for proceeding traditional male circumcision [17]. Therefore, public health intervention should focus on rearranging the rules on TMC standards practice, giving training to familiarize operation procedure and general hygiene techniques and the establishing of cross-referral systems between traditional circumcisers and health care givers.

The current study also applies multilevel generalized linear mixed model to determine factors associated with male circumcision. Accordingly, age, age at circumcision, place of residence, media exposure, sex of household head and age of household head were significantly associated with traditional male circumcision in Ethiopia at *p*-value 0.05.

The present study documented that the odds of traditional male circumcision were higher among male aged 20–59 than male aged less than 15–19 years. This implies that old male are more likely to be circumcised traditionally and the young get circumcised medically. This might be due to the establishment of the medical male circumcision services as Voluntary medical male circumcision (VMMC) for HIV prevention in health facilities in Ethiopia. Since 2007, VMMC was recommended by WHO and UNAIDS as a key component of combination HIV prevention in countries with a high HIV prevalence and low levels of male circumcision. Ethiopia was among the 14 countries which identified male circumcision as a priority and initiated programmes to expand the provision of male circumcision [18]. Our finding also suggested that the likelihoods of traditional male circumcision were low among male who were circumcised after the age of five than before the age of five.

As traditional male circumcision is usually associated with a religious or cultural ceremony [18], the present study also reported the significant association between religious status and TMC. Accordingly, the odds of traditional male circumcision were low among Christian male (orthodox and protestant) as compared with Muslim male. In this study, place of residence was also another factor that showed a significant association with the practice of traditional male circumcision. Rural male were more likely to be circumcised by traditional methods than urban male. This might be related to unavailability of nearby health facilities which give medical male circumcision service as in urban settings. Therefore, the public health intervention should focus on accessibility of medical male circumcision services for rural community.

Wealth index was also an important variable that showed a significant association with traditional male circumcision. The odds of traditional male circumcision were low among rich male as compared with poor male. This might be due to affordability of medical male circumcision service. Thus, the public health interventions should focus not only on accessibility of MMC but also on affordability of the service for all community members. Besides, the likelihoods of being circumcised traditionally were low among media exposed male than their counterparts. Therefore, we can also use different media as one route of intervention for community education and awareness creation; so that parents and adolescents are able to make informed choices about which services are safe, affordable and accessible to male circumcision.

### Strength and limitation of the study

The present study has some strengths. First, the study used nationally representative datasets and the estimates were performed after the data were weighted for sampling weight (men’s sample weight), primary sampling unit, and strata. Therefore, the results of this study are generalizable to the target population. Secondly, the current study has identified statistically significant hotspot areas of TMC using GIS and SaTScan soft wares. Thirdly, associated factors of TMC were determined using an advanced model (multilevel generalized linear mixed model) that accounts cluster nature of the data.

This study has also some limitations that should be kept in mind when making the conclusion. First, this study used self-reported circumcision status; this may be prone to either recall bias or social desirability bias. Secondly, the location of data values was shifted up to 2 km for urban and up to 5 km for rural areas.

## Conclusion

The spatial distribution of TMC was non-random. The primary clusters of TMC were identified in the southern part of Oromia, Tigray, northern part of SNNPR, Amhara, Gambella and Benishangul regions. Current age, age at circumcision, ethnicity, religion, place of residence, wealth index, media exposure, sex of household head and age of household head were factors associated with TMC in Ethiopia. Therefore, there is a need to rearrange the regulations on standards of TMC practice, arrange training to familiarize operation technique and general hygiene procedures and to launch cross-referral systems between traditional circumcisers and health workers. While undertaking these Public health interventions, due attention should be given for the identified clusters & significant actors.

## Data Availability

The datasets supporting the conclusions of this article are available upon request to the corresponding author.

## Abbreviations

AOR: Adjusted odds ratio
CI: Confidence interval
COR: Crude odd ratio
CSA: Central statistics agency
DHS: Demography heath survey
EAs: Enumeration areas
EDHS: Ethiopian demographic and health survey
HIV: Human immunodeficiency virus
SNNPR: Southern nation and nationality and people’s regions
TMC: Traditional Male Circumcision
VMMC: Voluntary medical male circumcision
WHO: World health organization
